# *Wabishki Bizhiko Skaanj*: a learning pathway to foster better Indigenous cultural competence in Canadian health research

**DOI:** 10.17269/s41997-020-00468-2

**Published:** 2021-05-18

**Authors:** Helen Robinson-Settee, Craig Settee, Malcolm King, Mary Beaucage, Mary Smith, Arlene Desjarlais, Helen Hoi-Lun Chiu, Catherine Turner, Joanne Kappel, Jonathon M. McGavock

**Affiliations:** 1 Wabishki Bizhiko Skaanj Learning Pathway, Can-SOLVE CKD Network, Vancouver, BC Canada; 2 Indigenous Peoples’ Engagement and Research Council, Can-SOLVE CKD Network, Vancouver, Canada; 3 Patient Governance Circle, Can-SOLVE CKD, Vancouver, Canada; 4 Can-SOLVE CKD, Vancouver, Canada; 5 Department of Community Health and Epidemiology, University of Saskatchewan, Saskatoon, SK Canada; 6 Saskatchewan Centre for Patient-Oriented Research, Saskatoon, Canada; 7 School of Nursing, Queen’s University, Kingston, ON Canada; 8 Patient-Centred Performance Improvement, BC Renal, Vancouver, Canada; 9 Division of Nephrology, Faculty of Medicine, University of Saskatchewan, Saskatoon, Canada; 10 Diabetes Research Envisioned and Accomplished in Canada (DREAM) theme, Winnipeg, MB Canada; 11 Department of Pediatrics and Child Health, Rady Faculty of Health Sciences, University of Manitoba, Winnipeg, Canada

**Keywords:** Indigenous health, Racism, Cultural competence, Canadian healthcare, Santé autochtone, racisme, compétence culturelle, soins de santé au Canada

## Abstract

**Objective:**

In Canada, Indigenous people experience racism across diverse settings, including within the health sector. This has negatively impacted both the quality of care that Indigenous people receive as well as how research related to Indigenous populations is conducted. Therefore, an Indigenous-led council at a kidney research network, in partnership with other key stakeholders, sought to create a learning pathway that aims to distill the racism that Indigenous people face, and build cultural competence, within the health sector.

**Participants:**

The learning pathway was designed for researchers, health care providers, patient partners and administrators.

**Setting:**

Various components of the pathway are established trainings in healthcare and research settings at provincial and national levels. Provincially, some components are implemented in British Columbia, Alberta, Saskatchewan, Manitoba and Ontario.

**Intervention:**

The pathway, called *Wabishki Bizhiko Skaanj* (meaning “White Horse” in Anishinaabemowin), involves six key steps: a culturally tailored blanket exercise that walks participants through the history of local Indigenous Nations/peoples; a more detailed online training program (San’yas); a series of webinars on Indigenous research ethics and protocols; an educational booklet about engaging Knowledge Keepers in research, as well as sharing details about their traditional knowledge and culture; two certification programs about Indigenous ownership of data; and a “book club,” wherein the conversation of racism—and the goal for finding solutions—is continually discussed.

**Outcomes:**

*Wabishki Bizhiko Skaanj* is working to build cultural competence in the Canadian health sector.

**Implications:**

This learning pathway has the potential to address racial disparities across the country and improve health outcomes for Indigenous peoples.

## Résumé

**Objectif:**

Au Canada, les autochtones font face au racisme dans divers milieux, y compris dans le secteur de la santé. Cela a eu une incidence négative sur la qualité des soins que reçoivent les peuples autochtones, ainsi que sur la façon dont la recherche sur les populations autochtones est menée. Par conséquent, un conseil dirigé par des Autochtones dans un réseau de recherche sur les reins, en partenariat avec d’autres parties prenantes, a cherché à créer une voie d’apprentissage visant à réduire le racisme auquel les peuples autochtones sont confrontés et à renforcer les compétences culturelles au sein du secteur de la santé.

**Participants:**

La voie d’apprentissage a été conçue pour les chercheurs, les fournisseurs de soins de santé, les patients partenaires et les administrateurs.

**Cadre:**

Cette voie d’apprentissage est composée de formations établies dans le milieu de la santé et de la recherche au niveau provincial et national. À l’échelle provinciale, il y a des programmes ayant lieu en Colombie-Britannique, en Alberta, en Ontario, en Saskatchewan et au Manitoba.

**Intervention:**

La voie d’apprentissage, appelée *Wabishki Bizhiko Skaanj* (qui signifie « cheval blanc » en Anishinaabemowin), comporte six étapes clés : un exercice culturel sur mesure qui guide les participants à travers l’histoire des nations/peuples autochtones locaux à l’aide de diverses couvertures culturelles; un programme de formation en ligne détaillé (San’yas); une série de webinaires sur l’éthique et les protocoles autochtones en matière de recherche; un livret pédagogique sur l’engagement des gardiens de la connaissance en recherche ainsi que le partage d’informations sur leur connaissance et leur culture traditionnelle; deux programmes de certification sur la propriété autochtone des données; et un « club de lecture » dans lequel nous ne cessons de discuter du racisme tout en cherchant des solutions.

**Résultats:**

*Wabishki Bizhiko Skaanj* s’efforce de renforcer les compétences culturelles dans le secteur de la santé au Canada.

**Implications:**

Cette voie d’apprentissage a le potentiel de s’attaquer aux disparités raciales à l’échelle nationale et d’améliorer les résultats en matière de santé pour les peuples autochtones.

## Introduction

Indigenous people in Canada have faced a long history of systemic racism, oppression and inequality that has negatively impacted their access to healthcare, quality of healthcare received, and participation and leadership in health research. These structural barriers and health inequities have persisted for generations in part because of many widespread misconceptions about Indigenous people among many non-Indigenous researchers and healthcare providers, as well as a lack of understanding of Indigenous peoples’ cultures, histories and experiences with colonialism; this lack of knowledge stems from racially constructed narratives about the superiority of colonial Canada and the inferiority of Indigenous ways of life (Wylie and Mcconkey 2018).

Unfortunately, racially biased misconceptions can negatively impact the quality of care Indigenous people receive—with sometimes fatal consequences (Geary 2017). In turn, racism can negatively impact Indigenous people’s feelings and trust towards the healthcare system, causing them to strategize around anticipated racism before visiting an emergency room or in some cases avoid seeking care altogether (Cameron et al. 2014) (Tang and Browne 2008).

Racial biases, as well as a pervasive bias for Westernized medicine in Canada, have dramatically impacted the design and conduct of health research related to Indigenous peoples in Canada. More critically, there is a pervasive lack of Indigenous representation within the healthcare and research setting. In the absence of Indigenous partners or scholars in health research, studies are often designed without adequate consultation of Indigenous peoples and/or do not incorporate Indigenous customs, culture or knowledge into the research process. This lack of access to health research and this promotion of settler-led research studies can perpetuate stereotypes and create barriers for Indigenous people in receiving appropriate care, contributing to the research process and maintaining autonomy over their own healthcare.

Importantly, there is good evidence to suggest that creating supportive healthcare environments that are inclusive of Indigenous people’s unique cultures, rights and perspectives will lead to health improvements (Richmond and Cook 2016). Advancing culturally safe *healthcare* reflects the need for more culturally safe health *research* to inform those practices and rebuild trust in health research among Indigenous peoples. Culturally safe research practices are those that create spiritually, socially, emotionally and physically safe environments for people in a cross-culture context.

Some critical steps that have been recommended to build cultural safety include reframing the conversation around race and health in Canada by acknowledging the foundational and ongoing realities of racism and colonialism; developing new interventions to address attitudinal, interpersonal and systemic racism towards Indigenous peoples; and implementing cultural safety training, which pays explicit attention to the power relations between service users and providers (Allan and Smylie 2015).

## The Wabishki Bizhiko Skaanj Learning Pathway

To help build cultural safety within the Canadian health sector, an Indigenous-led council at the Canadian kidney research network Canadians Seeking Solutions and Innovations to Overcome Chronic Kidney Disease (Can-SOLVE CKD), in partnership with stakeholders at the University of Manitoba Ongomiizwin Indigenous Research Institute and the Children’s Hospital Research Institute of Manitoba, has developed a Learning Pathway for researchers, healthcare providers, patient partners and administrators.

**Fig. 1:**
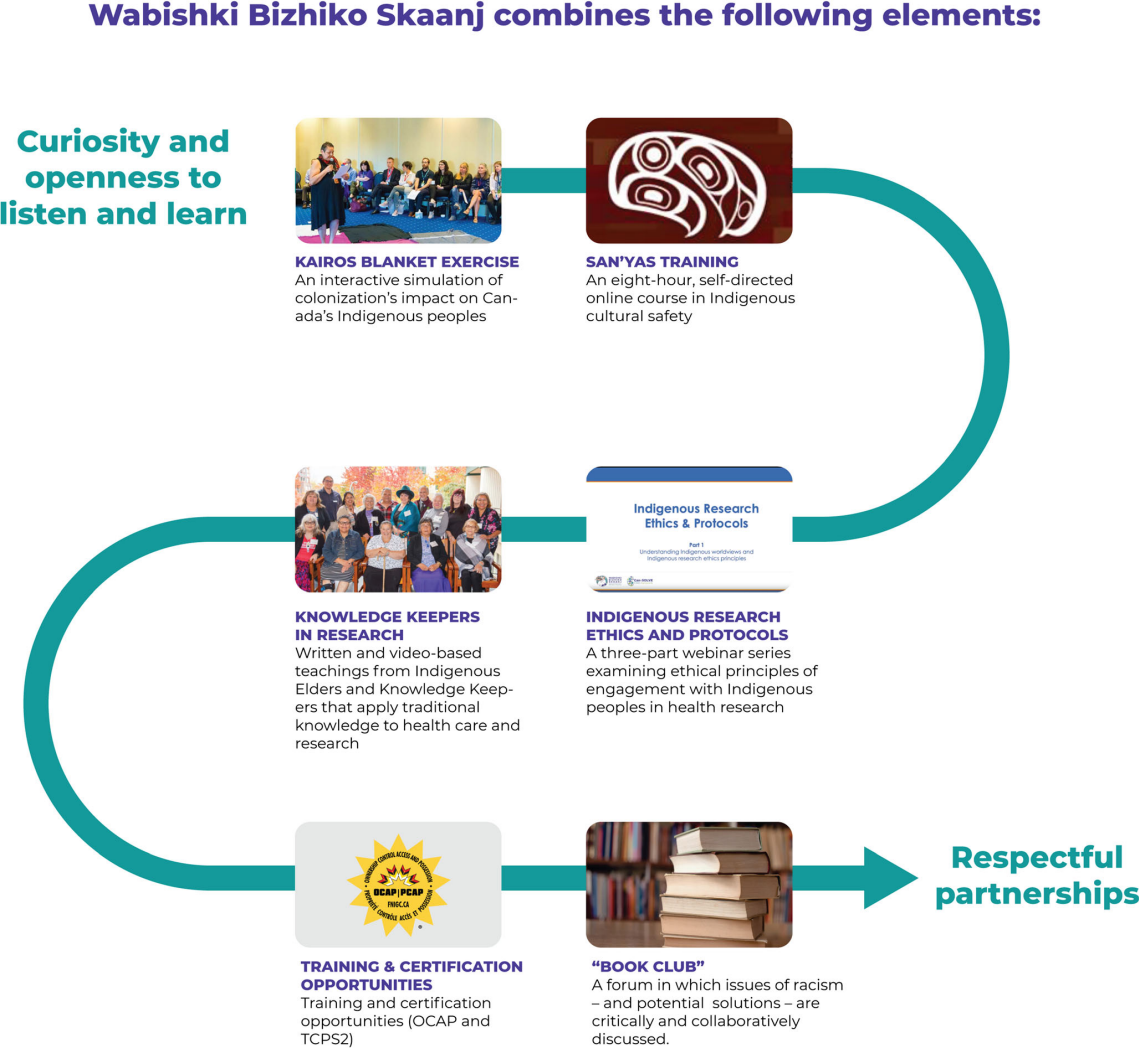
Wabishki Bizhiko Skaanj Learning Pathway diagram

The pathway, called *Wabishki Bizhiko Skaanj* (pronounced wah-bish-kih biish-ih-goo skaa-nch and meaning “White Horse” in Anishinaabemowin), involves strategically designed steps intended to help participants understand, recognize and distill racism that occurs within the health sector (Fig. 1). To do so, the learning pathway provides participants with more knowledge and awareness of racial biases, Indigenous voices and stories, the impact of colonization on Indigenous health, and culturally safe health research practices.

*Wabishki Bizhiko Skaanj* was designed and developed by members of an Indigenous-led council at Can-SOLVE CKD, called the Indigenous Peoples Engagement Research Council (IPERC). IPERC took a collaborative approach to identifying and designing the learning pathway through cross-country collaborations. In total, the learning pathway contains six key components, which are outlined in detail below and are at varying stages of development and implementation. Some components were created externally by other organizations and were incorporated within the learning pathway. Critically, there is much diversity among First Nations, Inuit, and Métis peoples across Canada, so each component of *Wabishki Bizhiko Skaanj* can be tailored to the appropriate local Indigenous group(s).

The pathway is designed to support people in learning about Indigenous cultures and histories; thus, it requires participants to partake in the various components of the programs with an open mind and willingness to bring about positive change. As participants engage in the learning pathway, they are encouraged to:

- *Look* within to observe and examine racial identities, privileges and biases;
- *Listen* to Indigenous voices and stories by participating in interactive learning exercises, facilitated online modules and webinars;
- *Learn* the history of colonization in Canada and its impacts on Indigenous peoples and their health;
- *Lead*, by reflecting on the learning and committing to taking appropriate actions in building genuine partnerships with Indigenous peoples and communities in the spirit of reconciliation.

Health organizations are welcome to adopt and implement any combination or all six of the components of* Wabishki Bizhiko Skaanj*, which are described below. While many of the components can be implemented by organizations using a top-down approach, two components—the Knowledge Keepers in Research and the webinar series on Indigenous Research Ethics and Protocols—are available on the Can-SOLVE CKD website to individuals interested in accessing them.

### The KAIROS™ Blanket Exercise

Indigenous peoples in Canada have a long and rich history extending back for time immemorial. Indigenous peoples have a strong oral tradition based on stories passed from generation to generation, as well as diverse languages and cultures that connect them to the Land and to the Creator. Knowledge Keepers are the holders of Indigenous knowledge and help to understand Indigenous ways of knowing, being and doing.

However, the way of life for Indigenous Nations/people was dramatically altered with the arrival of Europeans, with long-lasting, harmful impacts that remain a source of pain and trauma for many people today. These impacts include but are not limited to the slaughter of countless Indigenous people since first contact; the spreading of disease that killed millions; the Indian Act; the uprooting and relocation of many Indigenous groups; and the establishment of residential schools, whereby children were removed from their families, abused emotionally, physically and sexually, and forced to abandon their languages and cultures.

While many non-Indigenous people may be somewhat familiar with these experiences, it is important that major events in Indigenous history are better understood among the general public, providing more context to the ongoing trauma that persists to this day. This is especially important in the realms of healthcare and health research, where many physical or mental health complications of Indigenous people may be a direct result of colonialism-related traumas.

The KAIROS™ Blanket Exercise (KBE) program (Kairos Canada 2020) is a unique, participatory history lesson—developed by KAIROS Canada, an ecumenical non-profit organization for ecological justice and human rights, in collaboration with Indigenous Elders, Knowledge Keepers and educators—that fosters truth, understanding, respect and reconciliation among Indigenous and non-Indigenous peoples. It is a 90-min experiential workshop that aims to foster understanding about our shared history as Indigenous and non-Indigenous peoples.

During the KBE, participants walk on blankets representing the land and into the role of First Nations, Inuit and Métis peoples by reading scrolls and carrying cards which ultimately determine their outcome as they literally “walk” through situations that include pre-contact, treaty-making, colonization and resistance. Participants are guided through the experience by trained facilitators (who read the script and assume the roles of European explorers and settlers) and Indigenous Elders or Knowledge Keepers. The exercise concludes with a debriefing, conducted as a “talking circle,” during which participants discuss the learning experience, process their feelings, ask questions, share insights and deepen their understanding.

Since its creation in 1997, tens of thousands of KAIROS™ Blanket Exercises have been conducted in Canada and around the world.

### San’yas Indigenous cultural safety training

The second component of the *Wabishki Bizhiko Skaanj* learning pathway involves a seven-module course called San’yas Core Indigenous Cultural Safety Health Training. The online facilitated course delves deeper into the Indigenous history and culture that is brought to light with the blanket exercise.

While participants can complete the modules on their own time, they are also part of a discussion group with up to 25 other members. At the end of some modules, moderators will post related questions on the discussion board, which prompt participants to reflect on what they have learned. As well, participants are able to pose questions about the subject matter or ask for advice from other group members on how to foster cultural competence in their personal work environment.

San’yas was originally developed by the Provincial Health Services Authority Indigenous Health in British Columbia (BC) and captures the unique histories and cultures of First Nations/people in the province. However, additional province-specific versions of the program have since been created for Ontario and Manitoba, each of which capture the unique histories and cultures of relevant local Indigenous groups.

In Manitoba and BC, it is mandatory for employees of some health jurisdictions and departments to take San’yas training. As well, additional organizations are increasingly expressing interest in the tool and have voluntarily decided to implement it within their respective entities.

### Indigenous research ethics and protocols

The third component of the *Wabishki Bizhiko Skaanj* learning pathway delves into the ethical aspects of collaborating with Indigenous peoples in the research setting. Similar to the aforementioned components of the learning pathway, this component involves understanding the cultures and historical contexts of First Nations, Inuit and Métis peoples in Canada. However, it also highlights key ways in which the exchange of information and respect should flow reciprocally between Indigenous people and non-Indigenous people in research settings.

The starting premise of this component can be conveyed with the two-row wampum, a belt that symbolizes mutual respect and non-interference between Indigenous and non-Indigenous people. It emphasizes recognition that Indigenous and Western approaches are distinct and yet equally significant.

While this reciprocal flow of information and respect seems straightforward, it does not always occur in the research setting. In the past, Western academia has often involved the study of Indigenous peoples in a unidirectional manner, i.e., research “on” Indigenous peoples, not “with” (Smith 2012). As well, the traditional knowledge of Indigenous peoples has often not been given equal weight to the studies of academia. The third component of the learning pathway therefore aims to change this by fostering better recognition of the importance of traditional knowledge and inclusion of Indigenous peoples as equal partners in the research process. This is done through a three-part webinar series, with the themes of “Looking,” “Listening,” “Learning” and “Leading.”

The first webinar session helps participants *look* at the historical aspects of Indigenous research ethics, and it outlines current examples. The second session delves deeper in the topic of Indigenous research ethics and protocols by offering further territorial examples of community ethics and protocols, as well as institutional works. In this session, participants are encouraged to *listen* to what has been and is being done to ensure that community data and knowledges are not exploited. The final session helps participants to recognize *learnings* from these discussions and provides examples of how to continue being respectful of and incorporating Indigenous research ethics and protocols into practice. As a final parting message at the end of the webinar series, participants are encouraged to take what has been learned from these discussions and to lead by example in normalizing Indigenous research ethics and protocols into healthcare and research arenas.

Some important themes across all sessions include respect for First Nations, Inuit and Métis governing authorities; recognition of diverse interests within these communities; respect for community customs and codes of practice; review of research ethics before initiating any studies; and mutual research agreements among all parties.

### Engaging Knowledge Keepers in research

Knowledge Keepers are First Nations, Inuit and Métis who are chosen to hold important cultural and historical knowledge on behalf of their respective communities. “Elders (Knowledge Keepers) have a lot to contribute to research, but it’s up to the researcher to pay attention. You have to follow local protocol; research is learning, having knowledge – this is part of Indigenous culture” (October 2018. Dan Thomas. Personal interview with Helen Robinson-Settee. Manitoba).

The fourth component of the learning pathway encourages the inclusion and participation of Knowledge Keepers in research processes. To encourage this inclusion and help people understand the importance of Knowledge Keepers, the creators of the learning pathway convened Knowledge Keepers from across Canada and conducted a series of interviews. These interviews were conducted with two Knowledge Keepers from BC, one from Alberta, two from Saskatchewan, two from Manitoba, one from Ontario and one from Quebec. The interviews were transcribed and from the transcripts a narrative was created.

The narrative contains seven key strands: Essential Trainings/Learnings; Preparation for Meeting With a Knowledge Keeper; the Ask; Acknowledgement and Protocol; Knowledge; Who is a Knowledge Keeper?; and Relationships.

Members of IPERC are in the process of publishing this narrative as an online booklet and have initiated additional plans to create a video series of interviews with Knowledge Keepers. The multimedia and written materials will be available for health organizations and institutions to disseminate within their networks, to foster cultural competence and understanding of the importance of Knowledge Keepers among staff.

### Ownership, control, access and possession and Tri-Council Policy Statement-2 Certification

There are two existing certification programs that are recommended as part of the *Wabishki Bizhiko Skaanj* learning pathway. The first is the First Nations Principles of OCAP® (ownership, control, access, and possession). OCAP® asserts that First Nations have control over data collection processes in their communities and that they own and control how this information can be used. It also reflects First Nation commitments to use and share information in a way that maximizes the benefit to a community while minimizing harm.

As well, the learning pathway encourages use of the Tri-Council Policy Statement-2 Certification (TCPS-2) program, with an emphasis on Chapter 9 (Panel on Research Ethics 2018). This chapter is designed to serve as a framework for the ethical conduct of research involving Indigenous peoples. It is offered in a spirit of respect. It is not intended to override or replace ethical guidance offered by Indigenous peoples themselves. Its purpose is to ensure, to the fullest extent possible, that research involving Indigenous peoples is premised on respectful relationships. It also encourages collaboration and engagement between researchers and participants.

These two programs are important because they aim to help researchers and patient partners understand the principles of research and its elements from First Nations, Inuit and Métis perspectives, as well as from Western, colonial perspectives. This is in line with the concept of two-eyed seeing, whereby the knowledges of both Indigenous and non-Indigenous peoples are considered and respected (Martin 2012).

### A book club for continuing conversations

The journey towards a more culturally competent society does not stop upon completion of blanket exercises, training programs, webinars or certification programs—it is a continuous process. To continue the conversation about racism and culture competence within the healthcare system and beyond, IPERC recommends that people continually look, listen and learn from Indigenous stories outside of the learning pathway and help lead their communities towards a more competent healthcare and research environment. To support this final call to action, participants are encouraged to find resources capturing local Indigenous history and culture. Can-SOLVE CKD is considering publishing a recommended reading list on its website to support continuous learning.

## Discussion

Healthcare and research organizations are encouraged to adapt *Wabishki Bizhiko Skaanj* according to local Indigenous groups. As emphasized before, Indigenous groups across Canada have diverse histories, cultures and customs. For example, the Blanket Exercise can be done in different ways, according to the traditions of local Indigenous communities. Similarly, the historic context highlighted in the San’yas program should be tailored locally or regionally.

IPERC works with health organizations to implement the tool. Since the initial launch of *Wabishki Bizhiko Skaanj*, health organizations have increasingly expressed interest in the learning pathway and have committed to implementing components of it within their healthcare settings. For instance, the Canadian Society of Nephrology (CSN) and Diabetes Action Canada (DAC) have committed to rolling out some of the key components of *Wabishki Bizhiko Skaanj* in the coming years.

*Wabishki Bizhiko Skaanj* is a living pathway that can be further developed depending on the needs of healthcare professionals, researchers and patient partners. As the conversations around racism in the healthcare system evolve, so too can the learning pathway. IPERC, in partnership with the Learning Pathway working group, plans on continuing its efforts to develop and evaluate the tool. For example, the working group and an evaluation expert from the Centre of Excellence on Partnership with Patients and the Public (CEPPP) created a logic model, to support the working group in developing evaluation tools for each component of the learning pathway, as well as overall experience/satisfaction of the learning pathway as a whole.

## Conclusion

Significant disparities remain in terms of the access and quality of healthcare that Indigenous people in Canada experience as compared with non-Indigenous people. Major underlying causes include widespread misconceptions about Indigenous people among healthcare workers and researchers, as well as a lack of understanding of Indigenous peoples’ cultures, histories and experiences with colonialism. *Wabishki Bizhiko Skaanj* is a learning pathway that is adaptable for healthcare and research organizations to distill racism and boost cultural competence within their networks. This learning pathway offers a promising way to curtail racism in health and advance cultural safety in health research and care.
